# Work scheduling through communication tools and job satisfaction: The roles of work-family conflict and perceived work pressure

**DOI:** 10.1371/journal.pone.0352350

**Published:** 2026-07-06

**Authors:** Zhengxiu Sun, Senhu Wang, Yaguang Yan, Hong Tan, Shuangshuang Song, Shuanglong Li

**Affiliations:** 1 School of Economics and Management, Southeast University, Nanjing, China; 2 Department of Sociology and Anthropology, National University of Singapore, Singapore, Singapore; 3 Graduate School of Arts and Letters, Tohoku University, Sendai, Japan; 4 Department of Economics and Management, Tohoku University, Sendai, Japan; 5 School of Public Administration, Sichuan University, Chengdu, China; 6 College of Public Finance and Investment, Shanghai University of Finance and Economics, Shanghai, China; 7 Business School, National University of Singapore, Singapore, Singapore; 8 School of Social Development, East China University of Political Science and Law, Shanghai, China; Ningbo University, CHINA

## Abstract

The expansion of digital communication has increasingly blurred the boundaries between work and personal life, making work scheduling through communication tools more common. In China, mobile platforms such as WeChat are widely used to facilitate work-related scheduling and coordination, allowing work instructions and messages to be communicated without being confined to fixed time periods. Using nationally representative data from the 2021 China General Social Survey (CGSS), this study examines the association between communication-tool-mediated work scheduling and employees’ job satisfaction. We use multiple linear regression models to assess whether respondents’ work could be scheduled at any time via WeChat or phone and whether this workplace practice is associated with job satisfaction. Then, we further examine the mediating roles of work–family conflict and perceived work pressure using the KHB method, and conduct supplementary subgroup analyses by socioeconomic status (SES). The results show that work scheduling through communication tools is negatively associated with job satisfaction, and that this relationship is largely mediated by higher levels of work–family conflict and perceived work pressure. In addition, SES-based subgroup regressions provide suggestive evidence that this negative association is mainly observed among individuals with higher educational attainment and those in professional occupations. These findings document how work scheduling through communication tools is related to employees’ subjective evaluations of work and highlight heterogeneity in these associations across socioeconomic groups in contemporary Chinese workplaces.

## 1. Introduction

The COVID-19 pandemic accelerated the integration of digital communication technologies into everyday work routines, further weakening long-standing boundaries between work and personal life [1]. In China, platforms such as WeChat and mobile phones became indispensable not only for maintaining organizational functioning during lockdowns, but also for enabling communication-tool-mediated work scheduling—that is, the delivery of work instructions, coordination requests, and follow-up messages at any time through digital communication tools [2]. What emerged as an emergency practice has since evolved into a normalized mode of managerial coordination, potentially making work arrangements less confined to conventional temporal boundaries. This form of technology-enabled intrusion blurs temporal boundaries and introduces new sources of psychological strain. These trends raise pressing questions about how communication-tool-mediated work scheduling affects workers’ job satisfaction, and whether such effects are experienced equally across different socioeconomic groups in the post-pandemic labor landscape.

Although research on digital labor and technology-mediated work has grown rapidly, existing studies remain overwhelmingly rooted in Western contexts [3–5]. Much of this literature focuses on formal teleworking arrangements and enterprise communication systems situated within institutional environments that explicitly regulate the boundaries between work and private life—often supported by organizational norms and “right to disconnect” policies. The Chinese workplace differs fundamentally from this model. Communication apps such as WeChat, rather than designated work systems, have become deeply embedded in everyday organizational coordination, effectively merging social and professional spheres. Its multifunctionality and informality normalize communication-mediated work scheduling, while workplace norms surrounding authority, responsiveness, and collective obligation may make declining or delaying work-related requests socially costly. Despite this distinctive socio-technical configuration, empirical research on how such informal, communication-tool-mediated work contact shapes workers’ psychological experiences remains limited—especially studies drawing on nationally representative data. Likewise, there is little theoretical or empirical work explaining why these effects occur or whether they vary across socioeconomic status (SES) groups.

Given this context, this study examines how communication-tool-mediated work scheduling is associated with employees’ job satisfaction in China. Drawing on nationally representative data from the 2021 China General Social Survey (CGSS), we use multiple linear regression models to estimate the association between being assigned work-related tasks at any time via WeChat or phone and overall job satisfaction. To elucidate the underlying processes, we apply the Karlson–Holm–Breen (KHB) method to decompose total effects into direct and indirect components. Specifically, we test two mediating mechanisms—work–family conflict (WFC) and perceived work pressure—through which communication-tool-mediated work scheduling may alter employees’ evaluations of their jobs. Finally, we assess whether these effects vary across SES subgroups, with particular attention to differences by education and occupational class.

This study advances research on digital labor, technology-mediated work, and the work–family interface in three main ways. First, it moves beyond the broad literature on digital availability and well-being by focusing on a more specific practice—being assigned work tasks at any time through communication tools. This focus allows us to distinguish communication-tool-mediated work scheduling from general digital connectivity and to show how such practices may involve implicit labor input and non-wage job disutility that are only weakly captured by formal working-time arrangements. Second, drawing on Boundary Theory and the Job Demands–Resources (JD-R) framework, this study clarifies the psychosocial pathways linking communication-tool-mediated work scheduling to lower job satisfaction. By showing that WFC and perceived work pressure account for an important part of the observed relationship, the analysis provides empirical evidence on how digitally mediated work demands are translated into workers’ subjective evaluations of their jobs. Third, by conducting SES-based subgroup regressions, this study provides suggestive evidence on where the negative association is most clearly observed, particularly among employees with higher educational attainment and those in professional occupations, thereby offering preliminary insights into how communication-tool-mediated work scheduling may be experienced differently across socioeconomic positions. Taken together, these contributions extend current research beyond the general correlational framework of digital connectivity and well-being, and position communication-tool-mediated work scheduling as an important issue for the study of informal labor demands, boundary control, and labor governance in the digital era.

## 2. Literature and research hypotheses

### 2.1. Communication-tool-mediated work scheduling and job satisfaction

While a substantial body of research has examined the expansion of digital work—the performance of occupational tasks through information and communication technologies—far less attention has been devoted to the temporal dimension of digitalization: how work is scheduled, coordinated, and delegated through online communication channels. This distinction is analytically important. Digital work concerns the technological mediation of what employees do, whereas work scheduling through electronic communication channels concerns how employees may be contacted, assigned tasks, and expected to remain available and responsive across time. The latter directly shapes workers’ temporal autonomy, expands managerial reach beyond fixed working-time boundaries, and increases the permeability of work–life boundaries. Because temporal control is a core element of job quality, online communication-based work scheduling—especially when it stretches beyond regular working hours—is likely to exert a significant influence on employees’ subjective evaluations of their jobs, including their overall job satisfaction.

In this study, communication-tool-mediated work scheduling refers to the assignment, coordination, or adjustment of work tasks through social or mobile communication platforms—such as WeChat or phone calls—within and beyond regular working time. Unlike formal scheduling mechanisms that operate through standardized procedures, this form of work scheduling is enacted through socially embedded, informal, and often asynchronous communication. This mode of scheduling effectively extends managerial coordination across the temporal boundaries of employees’ work and personal lives. Although it may offer short-term flexibility or improve responsiveness, it also creates temporal uncertainty, heightens boundary permeability, and introduces new forms of psychological strain.

In contemporary work practice, the widespread adoption of digital communication technologies has increasingly blurred the once-clear spatial and temporal boundaries between work and personal life [6]. Platforms such as WeChat and mobile phones not only enhance the efficiency of information exchange and work coordination, but also make it easier for work arrangements to unfold through communication tools beyond fixed time boundaries, thereby weakening employees’ psychological detachment from work [7]. In this environment, work-related messages can easily extend into non-working time, eroding temporal autonomy and heightening the likelihood that employees’ personal time will be encroached upon by work.

Boundary Theory [8] offers a valuable lens for understanding how work scheduling via communication tools reshapes employees’ work experiences. The theory argues that individuals construct and maintain temporal, spatial, and psychological boundaries to minimize role interference and protect their well-being. When work intrudes into non-work domains or extends beyond fixed working-time boundaries through ongoing communication-based work coordination, these boundaries become increasingly permeable. Employees remain cognitively and emotionally connected to work during their personal time, reducing opportunities for recovery, limiting psychological detachment, and ultimately undermining their overall evaluations of work.

The erosion of boundaries is further intensified by the unstructured, immediate, and portable nature of mobile communication. When work-related messages, calls, and requests cross fixed working-time boundaries or extend into non-working time, the very notion of being temporally unavailable for work becomes ambiguous, leading to continuous role overlap and heightened fatigue. Such a state of persistent communication-enabled work reachability has been closely associated with technostress—the anxiety, cognitive overload, and adaptive pressures generated by constant and uncontrollable digital connectivity [9]. This strain not only disrupts work–family balance [10], but also undermines employees’ sense of autonomy and control, two core psychological resources that strongly shape overall job satisfaction.

Empirical research offers further support for these dynamics. Sustained expectations for digital availability have been linked to heightened anxiety, emotional volatility, and diminished temporal control, all of which reflect the strain produced by continuous connectivity [3]. These findings suggest that although digital scheduling may enhance organizational responsiveness, it can simultaneously erode employees’ temporal agency and autonomy, thereby undermining their overall job satisfaction. Building on this theoretical and empirical groundwork, we propose the following hypotheses:

**Hypothesis 1: Work scheduling through communication tools (e.g., WeChat or phone) is negatively associated with job satisfaction.**

### 2.2. The mediating roles of WFC and perceived work pressure

From the perspective of Boundary Theory [8], work scheduling through communication tools increases the permeability and instability of boundaries separating work and family roles. When task assignments and coordination occur through digital communication channels across or beyond standard working-time boundaries, the spatial and temporal segmentation that typically organizes daily life becomes blurred. This heightened permeability elevates the likelihood of WFC—a form of inter-role interference in which work-related expectations encroach upon personal or family responsibilities.

While digital communication may appear to offer flexibility, it simultaneously and often invisibly extends managerial and collegial coordination into employees’ non-work time and across fixed temporal boundaries. As a result, workers must navigate multiple and competing role expectations, often under conditions of temporal unpredictability. The expectation of remaining responsive to work requests through communication tools—particularly salient in workplace settings where responsiveness to work-related requests is strongly expected—reduces opportunities for psychological detachment and recovery, generating cumulative strain. Extensive empirical evidence shows that WFC undermines job satisfaction and overall life evaluation by depleting emotional energy and weakening role clarity [11–14]. Conversely, relational and institutional supports—such as family-supportive supervisory behaviors—can buffer these negative effects by facilitating more positive forms of work–family spillover [15].

The JD-R framework provides a complementary explanation for why communication-tool-mediated work scheduling may increase perceived work pressure and reduce job satisfaction. The JD-R framework distinguishes between job demands, which require sustained physical or psychological effort, and job resources, which help employees manage these demands and protect their well-being [16]. Communication-tool-mediated work scheduling may function as an additional job demand because it increases temporal unpredictability, requires employees to remain attentive to possible work instructions, and may reduce opportunities for recovery. When these demands are not matched by sufficient resources—such as temporal autonomy, control over work scheduling, or the ability to disengage from work—employees are more likely to experience heightened work pressure and evaluate their jobs less positively [17]. In this sense, Boundary Theory and the JD-R framework offer complementary insights: the former explains how work scheduling through communication tools increases the permeability of work–life boundaries, while the latter explains how this practice may intensify demands and deplete workers’ psychological resources.

More specifically, the unpredictability and persistent demands associated with work scheduling through communication tools—such as receiving work assignments or follow-up instructions via WeChat or phone outside fixed work routines or beyond the immediate workplace context—often generate strain-based role conflict [18]. Employees must continually reallocate attention and cognitive resources to accommodate unexpected directives, a process that depletes mental energy and fosters cumulative fatigue. Unlike structured, face-to-face scheduling, informal and digitally mediated task delegation introduces a heightened form of temporal uncertainty, in which the timing, duration, and urgency of work obligations become difficult to anticipate or control. As work time becomes increasingly permeable, communication technologies function not only as coordination tools but also as instruments of continuous supervision and implicit accountability, reinforcing a persistent sense of availability and performance pressure.

Empirical evidence reinforces these dynamics. Studies employing the Minnesota Satisfaction Questionnaire (MSQ) show that elevated work pressure is negatively associated with intrinsic aspects of job satisfaction, including satisfaction with “the work itself” and with “supervisory practices” [19,20]. More recent research identifies digital overload anxiety—the tension provoked by sustained connectivity through digital communication—as a significant predictor of heightened work pressure and reduced job satisfaction [3]. Collectively, these findings indicate that communication-based work connectivity not only erodes the boundaries surrounding personal time but also intensifies employees’ psychological demands, ultimately weakening their overall evaluations of their work.

Accordingly, the foregoing discussion suggests that work scheduling through communication tools may be negatively associated with employees’ job satisfaction through multiple psychosocial pathways—most notably by increasing WFC and elevating perceived work pressure. This reasoning leads to the following hypotheses:

**Hypothesis 2A: WFC mediates the negative relationship between communication-tool-mediated work scheduling and job satisfaction.**
**Hypothesis 2B: Perceived work pressure mediates the negative relationship between communication-tool-mediated work scheduling and job satisfaction.**

### 2.3. SES-based heterogeneity in the association between communication-tool-mediated work scheduling and job satisfaction

Although work scheduling through communication tools may carry broad implications for workers, its effects are unlikely to be uniform across the labor force. Much existing research implicitly treats communication-mediated coordination as an equalizing force, assuming that all workers experience similar conditions. In practice, however, individuals’ ability to cope with—or resist—work demands conveyed through communication tools is deeply embedded within organizational hierarchies and broader class structures. SES captures these structural asymmetries by shaping not only workers’ differential exposure to digital coordination but also their access to resources, autonomy, and authority in defining the temporal boundaries of their work [21]. Examining SES-based differences therefore reveals how work scheduling through communication tools may reproduce or even intensify existing inequalities in temporal control and subjective well-being.

For high-SES employees, autonomy is a central component of their professional identity. Work scheduling through communication tools undermines this autonomy by imposing unpredictable demands on workers’ time. Even when workers internalize norms of constant availability, such norms do not eliminate strain; rather, they intensify it. Internalized expectations create identity dissonance—the tension between valuing autonomy and being unable to exercise it in practice. This dissonance, combined with high cognitive demands and limited temporal control [22], produces stronger boundary strain and greater declines in job satisfaction among high-SES workers [23–28].

In contrast, low-SES employees generally work in more routinized roles with clearer temporal structures and lower exposure to persistent work scheduling through communication tools. When such communication-based work intrusions do occur, they face them with weaker structural power—limited discretion, little capacity to negotiate timing, and heightened job insecurity [29]. Under these conditions, such requests are understood primarily as external constraints, not as violations of an autonomy-based professional identity. Compliance is therefore driven by necessity rather than by internalized norms of availability. Consequently, while work scheduling through communication tools still undermines their well-being, its subjective impact is less severe than for high-SES employees, whose autonomy-oriented habitus sustains a more permeable boundary between work and non-work domains [30]. We therefore propose the following hypothesis:

**Hypothesis 3: The negative association between communication-tool-mediated work scheduling and job satisfaction may be particularly evident among higher-SES workers.**

## 3. Methods

### 3.1. Data and sample

This study uses data from the 2021 wave of the China General Social Survey (CGSS). The data were obtained from the official CGSS data archive and were fully anonymized prior to release. The authors had no access to direct or indirect identifiers of individual participants during or after data collection. Since 2003, the CGSS has employed a multistage stratified probability-proportional-to-size random sampling approach to collect a nationally representative sample of the Chinese population [31]. To account for selection probabilities and non-response bias, all analyses were conducted using survey weights. Several steps were undertaken to construct the final analytical sample. First, we began with the full CGSS 2021 sample (N = 8,148). Second, we restricted the sample to individuals who responded to the key question: “In the past month, has your work been scheduled at any time via WeChat or phone?” (N = 1,973). Third, we further refined the sample by excluding respondents who selected “Not applicable” or “Don’t know” for key variables, resulting in a subsample of 1,576. Finally, considering the statutory retirement ages in China—65 for men and 60 for women—we applied an age restriction, limiting the sample to males under 65 years old and females under 60 years old. This resulted in a final analytical sample of 1,504 cases. The detailed steps of the sample selection process are presented in Table A1 in the appendix.

### 3.2. Measures

The dependent variable, job satisfaction, is measured by respondents’ self-reported evaluation of their current job, based on the CGSS item “Overall, how satisfied are you with your job?” Responses are coded on a five-point ordinal scale from 1 (“very dissatisfied”) to 5 (“very satisfied”), where higher values indicate greater satisfaction.

The independent variable, communication-tool-mediated work scheduling, is measured by a CGSS item asking whether respondents’ work can be scheduled at any time via WeChat or telephone (1 = yes, 0 = no).

The mediators in this study are perceived work pressure and WFC. Perceived work pressure was measured using the question, “How often do you experience work pressure in your workplace?”, with responses ranging from 1 (“rarely”) to 4 (“always”). This item is interpreted as a global indicator of respondents’ perceived work pressure rather than as a comprehensive multi-item scale of occupational stress or psychological strain. WFC was measured using two questions: “In general, do you feel that your work interferes with your family life?” and “Overall, do you feel that your family life gets in the way of your work?” Both items were rated on a scale from 1 (“never”) to 5 (“often”). We constructed the WFC score by averaging the responses to the two items. The two items showed acceptable internal consistency, with a standardized Cronbach’s alpha of 0.707 and an inter-item correlation of 0.547. Higher scores indicate greater perceived work pressure and stronger WFC, respectively.

We used two variables to examine subgroup variation, including educational level (secondary and below, tertiary and above) and occupation status (non-professional, professional). Educational level was classified according to respondents’ highest completed level of education. The “secondary and below” group included respondents with no formal education, literacy-class or private-school education, primary education, junior secondary education, vocational junior secondary education, general senior secondary education, or secondary vocational education. The “tertiary and above” group included respondents with technical-school, college, university, or postgraduate education. Occupational status was constructed using respondents’ current occupation codes, employment status, and supervisory responsibilities. Current occupations, originally coded according to ISCO-08, were converted into ISCO-88 occupation codes, and information on self-employment status and supervisory responsibility was incorporated using the EGP occupational-class framework. Respondents in professional or managerial occupations were classified as professional/managerial workers, while respondents in routine non-manual, manual, and farm-labor occupations were classified as non-professional workers.

Additionally, we controlled for a number of demographic, household, and work characteristics that prior research has shown to affect workers’ job satisfaction [32–34]. These covariates include gender, age, age squared, *hukou* type, educational level, marital status, parenthood status, union membership, party membership, region type, occupation, working hours per week, and working years in current job. The weighted descriptive statistics for all variables used in the analysis are available in Table 1.

**Table 1:** Weighted descriptive statistics of key variables.

Dependent variables	
Job satisfaction (1–5), M (SD)	3.60 (0.85)
**Independent variable**	
Communication-tool-mediated work scheduling, %	
Yes	75.12%
No	24.88%
**Control variables**	
Gender, %	
Male	56.56%
Female	43.44%
*Hukou* type, %	
Agricultural	50.62%
Non-Agricultural	24.56%
Others	24.82%
Educational level, %	
Secondary and below	64.78%
Tertiary and above	35.22%
Marital status, %	
Never married	20.17%
Married/cohabiting	73.69%
Divorced/separated/widowed	6.14%
Union member, %	
Yes	19.20%
No	80.80%
Parenthood status, %	
Yes	74.94%
No	25.06%
Party membership, %	
Yes	11.88%
No	88.12%
Occupation, %	
Professional	36.65%
Non-professional	63.35%
Region type, %	
Western	15.49%
North East	6.80%
Central	28.95%
East	48.76%
Age, M (SD)	38.91 (10.61)
Working hours per week, M (SD)	50.94 (16.73)
Working years in present job, M (SD)	7.76 (7.90)
**Mediating variables, M (SD)**	
WFC (1–5), M (SD)	1.79 (0.82)
Perceived work pressure (1–4), M (SD)	1.94 (0.96)
N (observations)	1,504

*Note.* Percentages (%) are shown for categorical variables and means (M) and standard deviation (SD) are shown for continuous variables. All results are weighted using the sampling weights. Region type follows the four-region classification used by the National Bureau of Statistics of China. The classification is used to capture broad regional contextual differences rather than as a direct measure of economic development or urbanisation.

### 3.3. Analytic strategy

The analysis proceeded in three stages. In the first stage, we estimated baseline models to examine how work scheduling through communication tools is associated with job satisfaction, using multiple linear regression. To ensure that the estimated effects were not confounded by individual or structural characteristics, we included a comprehensive set of control variables capturing demographic, occupational, and contextual factors: gender, age, marital status, parenthood, *hukou* type, education, party and union membership, occupation, region type, weekly working hours, and job tenure. These covariates account for differences in personal attributes, employment arrangements, and regional contexts that may jointly shape employees’ job satisfaction. The baseline specification is given by:


$$\begin{array}{c} {Job\;Satisfaction}_{i}\;=\;\beta_{\text{0}}\;+\;\beta_{\text{1}}\;{Communication}_{i}\;+\;X_{i}\;\beta \;+\;\epsilon_{i} \end{array}$$

where ${Job\;Satisfaction}_{i}$ serves as the dependent variable, while ${Communication}_{i}$, i.e., communication-tool-mediated work scheduling, is the key independent variable of interest. The coefficient of primary interest is $\beta_{\text{1}}$, which captures the association between communication-tool-mediated work scheduling and employees’ job satisfaction. $X_{i}\;$ denotes a vector of control variables. $\epsilon_{i}$ is the error term.

In the second stage of the analysis, we examined the mechanisms through which work scheduling through communication tools shapes job satisfaction. Following Kohler et al. [35], we employed the KHB decomposition method, mainly as a convenient tool for decomposing the total effect of the key explanatory variable into direct and indirect components when considering multiple potential mediators simultaneously [36–38]. As the decomposition reflects statistical rather than causal pathways, the KHB approach helps us assess the extent to which WFC and perceived work pressure mediate the association between work scheduling through communication tools and job satisfaction. We do not regard KHB as the only or necessarily superior approach to mediation analysis. To assess whether the proposed statistical pathways are sensitive to the choice of method, we additionally conducted conventional two-/three-step mediation tests, Sobel tests, and bootstrap tests for each mediator separately.

In addition, we conducted further analyses across SES groups, focusing on two SES-related dimensions: educational attainment and occupational status. These analyses were intended to examine where the observed association between communication-tool-mediated work scheduling and job satisfaction was most clearly present.

## 4. Results

Table 1 reports the weighted descriptive statistics for all variables used in the analysis, including the dependent, independent, mediating, and control variables. The mean level of job satisfaction is 3.60 on a five-point scale, indicating a moderate degree of satisfaction among respondents. With respect to the main explanatory variable, approximately 75% of participants report that their work can be scheduled via WeChat or mobile phone, highlighting the widespread prevalence of work scheduling through communication tools in contemporary Chinese workplaces. This high prevalence should be interpreted as an imbalance in exposure rather than as a conventional ceiling effect, as approximately one quarter of respondents remain in the unexposed category and therefore provide a meaningful basis for comparison. Nevertheless, because the measure is binary, it cannot capture variation in the frequency or intensity of such scheduling practices.

### 4.1. Baseline results

Table 2 reports the regression results examining the association between communication-tool-mediated work scheduling and job satisfaction. Model 1 includes only the focal independent variable and shows a significant negative relationship (coef. = –0.086, p < 0.05). Model 2 adds the full set of control variables, and the association remains negative and statistically significant (coef. = –0.100, p < 0.05). To account for selection probabilities and potential non-response bias, Model 3 applies survey weights; the coefficient becomes slightly more pronounced (coef. = –0.115, p < 0.05). Model 4 excludes respondents beyond China’s statutory retirement ages to ensure that the analysis reflects the active labor force, yielding a substantively similar estimate (coef. = –0.117, p < 0.05). Full estimates for all covariates are provided in Table A2 in the appendix. Taken together, these results provide consistent support for Hypothesis 1, indicating that communication-tool-mediated work scheduling is negatively associated with job satisfaction.

**Table 2:** The association between communication-tool-mediated work scheduling and job satisfaction.

	Job satisfaction			
	Model 1	Model 2	Model 3	Model 4
Communication-tool-mediated work scheduling	−0.086**	−0.100**	−0.115**	−0.117**
	(0.043)	(0.048)	(0.057)	(0.058)
Control variables	No	Yes	Yes	Yes
Observations	1,967	1,576	1,576	1,504
R-squared	0.002	0.082	0.093	0.088

*Note*: Model 2, 3, and 4 control for gender, age, age squared, *hukou* type, educational level, marital status, parenthood status, union status, party membership, region type, occupation, working hours per week, working years in current job. Standard errors in parentheses. * p < 0.1, ** p < 0.05, *** p < 0.01.

To ensure the robustness of the above findings, we further conducted the following robustness checks. First, given that job satisfaction is reported on a five-point scale, we use OLS as the primary specification to facilitate direct interpretation of the estimated coefficients in score units. To account for the ordinal nature of the outcome variable, we further re-estimated the baseline specification using ordered logit and ordered probit models. As shown in Appendix Table A3, the estimated association remains negative and statistically significant in both specifications, suggesting that the main finding is not sensitive to the choice of model specification.

Second, to examine whether potential non-random response to the key survey item affects our findings, we employed the Heckman two-step procedure [39,40] as a robustness check for possible sample selection bias. Specifically, the first-stage selection equation was estimated using the full sample, with a dummy variable indicating whether the respondent answered the question on communication-tool-mediated work scheduling as the dependent variable. We included an indicator for evening interview as an exclusion restriction, together with the individual and work-related control variables. This selection variable was excluded from the second-stage outcome equation, where job satisfaction was regressed on communication-tool-mediated work scheduling and the control variables. The results in Table A4 show that, both for the full sample and for the corresponding sample excluding respondents above statutory retirement age, the inverse Mills ratio (lambda) is not statistically significant, suggesting that there is no strong evidence of substantial sample selection bias. Importantly, the coefficient of the key explanatory variable remains negative and statistically significant, indicating that correcting for potential non-random item response does not materially alter the main conclusions.

Third, to assess whether the baseline association is sensitive to potential endogeneity concerns, we further employ the heteroskedasticity-based instrumental variable (IV) approach proposed by Lewbel [41]. The Lewbel approach exploits heteroskedasticity in the model to construct internal instruments and provides a feasible strategy for addressing potential endogeneity when suitable external instruments are unavailable. Its core idea is to generate instruments by interacting mean-centered exogenous variables with the first-stage residuals. The Breusch-Pagan test confirms significant heteroskedasticity in the model (LM = 37.058, p = 0.0052), satisfying the prerequisite for constructing internal instruments. The second-stage estimate remains negative and similar in magnitude to the baseline result, with statistical significance at the 10% level (coef. = −0.0948, p = 0.081). The weak-instrument diagnostics are reassuring, with both the Cragg–Donald Wald F statistic (709.497) and the Kleibergen–Paap rk Wald F statistic (872.039) exceeding the Stock–Yogo critical value for 10% maximal IV size (55.15), and the Hansen J test does not reject the overidentifying restrictions (J = 22.934, p = 0.1155). Given the cross-sectional nature of the data, we interpret this analysis as a supplementary robustness check rather than as definitive evidence of a causal relationship.

In addition, an interesting and important question is whether the above results mainly reflect a general requirement of standby availability, or instead the assignment of work tasks through communication tools. To examine this issue, we conducted a heterogeneity analysis based on the survey item, “In the past month, has your job required you to be on call by phone?” The results in Table A5 show that the findings reported in Table 2 are mainly concentrated among workers whose jobs do not require them to be on call by phone, while the effect is not statistically significant among those whose jobs already require phone on-call availability. This suggests that the negative effect identified in this paper cannot be simply understood as the result of a general standby requirement. Rather, the findings indicate that the focal measure captures a distinct work-arrangement practice: the possibility that work may be scheduled through communication tools without being confined to fixed time periods, including among workers whose jobs do not formally require phone on-call availability. For these workers, such scheduling may generate greater temporal uncertainty and may be more likely to disrupt established boundaries between work and non-work life.

### 4.2. Mechanism analysis

Table 3 reports the results of the KHB decomposition analysis, which assesses whether the proposed mediators account for the association between work scheduling through communication tools and job satisfaction. The indirect effect is significant and explains nearly the entire relationship (coef. = –0.125, p < 0.05; % mediated = 94.73), while the direct effect is not statistically significant. The decomposition further indicates that WFC and perceived work pressure both function as significant mediators. WFC accounts for 53.70% of the total effect (coef. = –0.067, p < 0.01), and perceived work pressure accounts for 41.04% (coef. = –0.051, p < 0.01). These results provide strong support for Hypotheses 2A and 2B, indicating that WFC and perceived work pressure statistically account for a substantial proportion of the association between work scheduling through communication tools and job satisfaction.

**Table 3:** KHB decomposition examining the mediation effects.

	Job Satisfaction	
Communication-tool-mediated work scheduling		
Total	−0.125**	
	(0.056)	
Direct	−0.007	*5.27%*
	(0.057)	
Indirect	−0.119***	*94.73%*
	(0.020)	
Decomposition of indirect effect (Coef., %)		
WFC	−0.067***	*53.70%*
	(0.015)	
Perceived work pressure	−0.051***	*41.04%*
	(0.014)	
Observations	1,494	
R-squared	0.18	

*Note.* The KHB analysis is based on Model 4 of Table 2. The lower number of observations compared to Model 4 is due to missing values in the mediating variables. When Model 4 is re-estimated using the same 1,494 observations as the KHB analysis, the coefficient of communication-tool-mediated work scheduling is −0.125. All models control for gender, age, age squared, *hukou* type, educational level, marital status, parenthood status, union status, party membership, region type, occupation, working hours per week, working years in current job. The contribution of each mediator to the total effect is shown in italics. Standard errors in parentheses. * p < 0.1, ** p < 0.05, *** p < 0.01

Since KHB is not the only available method for mediation analysis, we further supplement the analysis with two-/three-step mediation tests, Sobel tests, and bootstrap mediation tests (1,000 replications) as robustness checks; the corresponding results are reported in Appendix Table A6. The supplementary analyses yield consistent conclusions regarding the direction and statistical significance of the two proposed pathways.

### 4.3. Further analysis

To further examine SES-related patterns, we conducted subgroup analyses across SES groups, focusing on two dimensions: educational attainment and occupational status. As shown in Table 4, the estimated negative association between communication-tool-mediated work scheduling and job satisfaction is most clearly observed among individuals with higher educational attainment and those in professional occupations. By contrast, the association is smaller and statistically insignificant among individuals with lower educational levels or those in non-professional occupations. Supplementary analyses further indicate that the prevalence of communication-tool-mediated work scheduling differs across educational and occupational groups, suggesting that exposure to this practice may itself be socially patterned. Because the subgroup estimates in Table 4 are based on within-group comparisons between exposed and unexposed respondents, they should be interpreted as suggestive evidence consistent with Hypothesis 3 rather than as definitive tests of between-group differences.

**Table 4:** The association between communication-tool-mediated work scheduling and job satisfaction across educational level and occupation.

	Job Satisfaction	
Panel A. Educational level		
	Lower	Higher
Communication-tool-mediated work scheduling	−0.073	−0.234**
	(0.072)	(0.094)
Observations	839	665
R-squared	0.091	0.090
Panel B. Occupation		
	Non-professional	Professional
Communication-tool-mediated work scheduling	−0.094	−0.158*
	(0.074)	(0.095)
Observations	876	628
R-squared	0.091	0.088

*Note.* Both panels control for gender, age, age squared, *hukou* type, educational level (excluding Panel A), marital status, parenthood status, union status, party membership, region type, occupation (excluding Panel B), working hours per week, working years in current job. Standard errors in parentheses. * p < 0.1, ** p < 0.05, *** p < 0.01.

## 5. Discussion

Unlike many Western workplaces—where communication is routed through formal enterprise systems and increasingly constrained by “right-to-disconnect” norms—the coordination of day-to-day work in China appears to be increasingly mediated through WeChat and mobile phones, thereby blurring social and professional spheres. More importantly, this shift is not merely a technological change in communication channels; it reflects a broader transformation in how managerial control is exercised and how work time is organized. Within this hybrid sociotechnical environment, work scheduling through communication tools allows work demands to move more flexibly across fixed temporal boundaries and fosters expectations of continued responsiveness. As a result, the issue at stake is no longer simply whether employees are contacted outside regular hours, but whether digital communication is reshaping workers’ temporal autonomy itself. In this sense, the spread of communication tool-based work scheduling speaks directly to contemporary debates on job quality, boundary erosion, and the governance of digital labor, making its implications for job satisfaction and inequality especially important to understand.

Drawing on nationally representative data from the 2021 CGSS, this study (1) estimates the association between work scheduling through communication tools and job satisfaction, (2) identifies WFC and perceived work pressure as key mediating mechanisms, and (3) conducts SES-based subgroup analyses that provide suggestive evidence on where this association is most clearly observed. Taken together, the findings reposition communication-tool-mediated work scheduling as a form of temporal governance rather than a simple tool of convenience. This workplace practice is associated with boundary permeability and psychological strain, and it raises governance issues that extend beyond wages and hours to the timing, modality, and normative expectations of work coordination through communication tools. These insights have implications for organizational practice, including clearer communication protocols across work-time boundaries, and for broader sociological debates on psychological well-being in digitally connected work environments.

First, our baseline results show that work scheduling through communication tools is significantly negatively associated with employees’ job satisfaction. More importantly, the supplementary heterogeneity analysis indicates that this pattern is unlikely to be driven simply by a general requirement of standby availability or formal on-call work. Rather, the underlying issue appears to lie in the way communication tools allow work arrangements to be communicated and adjusted without being confined to fixed temporal routines. In other words, the negative association identified in this study is less about standby work per se than about a mode of task delegation that may make the timing of organizational demands less predictable through WeChat, phone calls, and related communication channels. This also helps explain the broader ambivalence of digitalization. On the one hand, communication technologies such as WeChat and mobile phones enhance coordination and flexibility, allowing organizations to sustain connectivity across time and space—especially under remote or hybrid work arrangements. On the other hand, once these tools become routine channels for task delegation and supervisory contact, they may increase the permeability of the boundaries between work and non-work domains and erode the temporal boundaries necessary for rest and psychological detachment [42–44]. The central issue, therefore, does not stem from the technologies themselves but from the institutional and cultural embedding of their use. As work responsiveness through communication tools becomes normalized, the promise of flexibility is gradually transformed into an obligation of being reachable for work.

Second, the mediation analyses provide further insight into the observed association between work scheduling through communication tools and job satisfaction. The KHB results show that a substantial proportion of this association is statistically accounted for by WFC and perceived work pressure, which are both consistent with the psychological consequences of increasingly permeable temporal boundaries in communication-intensive work settings. From the perspective of Boundary Theory [8], communication-tool-based work scheduling may be associated with greater boundary permeability, weaker psychological detachment, and higher levels of strain. WFC and perceived work pressure may therefore capture important aspects of the relationship between communication-tool-based work scheduling and workers’ evaluations of their jobs. These findings are also consistent with the JD-R framework, in which communication-tool-mediated work scheduling may represent an additional job demand associated with temporal unpredictability and the consumption of psychological resources, particularly when employees have limited control over when work demands arise. More broadly, these findings suggest that communication-tool-based work scheduling is related to workers’ experience of time and boundary management in ways that may have implications for well-being.

Third, the SES-based subgroup analyses provide suggestive evidence that the negative association between communication-tool-mediated work scheduling and job satisfaction is most clearly observed, in the subgroup estimates, among employees with higher educational attainment and those in professional occupations. Supplementary analyses further show that these employees are more likely to report exposure to communication-tool-mediated work scheduling, suggesting that digital communication tools may be more deeply embedded in their everyday work routines. For employees in higher educational and professional positions, work is often closely organized around autonomy, expertise, and self-directed responsibility. Work scheduling through communication tools at any time may therefore function not only as an additional task demand, but also as a source of pressure on temporal control and professional boundaries [45]. Greater exposure to such practices may also make work-related communication appear as a normalized part of professional responsibility, thereby reducing opportunities for psychological detachment from work. In this sense, the observed subgroup patterns suggest that communication-tool-mediated work scheduling may be especially relevant to understanding job satisfaction among employees in education- and occupation-based higher-SES positions.

At the same time, lower-SES employees are more likely to work in routinized jobs with clearer external supervision, where work organization is less centered on autonomy and self-management [46]. In such settings, work assignments conveyed through communication tools may be perceived primarily as organizational directives rather than as distinctive intrusions into autonomy-based professional boundaries. At the same time, these employees may have relatively limited bargaining power to negotiate or resist additional work demands [47]. Some work effort extended into non-work time through digital communication tools may therefore operate as implicit expectations or default responsibilities that are not clearly specified in formal contracts [48]. From this perspective, the absence of statistically significant associations among lower-SES workers does not necessarily mean that they are less affected in any objective sense; rather, it highlights the need for future research to examine how such practices are embedded in different forms of work organization.

The findings of this study have important implications for employee well-being and labor governance in communication-intensive workplaces. First, organizations should establish clearer norms regarding when work tasks may be assigned, through which communication channels they should be conveyed, and what forms of responsiveness are reasonably expected. In addition, such practices may involve additional work-related effort that is not fully reflected in formal working-time arrangements or clearly recognized in existing compensation structures. This suggests that the issue is not only one of employee well-being, but also one of informal labor demands, compensating differentials, and the adequacy of existing regulatory frameworks in digitally mediated work settings. Second, because the negative association identified here is linked to heightened WFC and perceived work pressure, management practices should pay more attention to workers’ temporal autonomy and psychological detachment, rather than focusing solely on efficiency and connectivity. Third, labor governance should remain attentive to the possibility that the consequences of work scheduling through communication tools may vary across the labor force. More broadly, these findings suggest that governance in the digital era must extend beyond wages and formal working hours to include the timing, modality, and boundary implications of communication-tool-based work assignment.

This study still has several limitations. First, the cross-sectional design does not allow us to establish temporal ordering or make rigorous causal inferences. Reverse causality is possible, as employees with lower job satisfaction may be more likely to perceive communication-tool-mediated work scheduling as intrusive or burdensome. Although the Lewbel IV analysis provides a supplementary sensitivity check when the focal variable is treated as potentially endogenous, it cannot definitively rule out reverse causality. Second, communication-tool-mediated work scheduling is measured using a single binary item, and perceived work pressure is measured using a single global item. These measures cannot fully capture the timing, frequency, intensity, or multidimensional psychological consequences of communication-based work demands. Future research should use more detailed and validated multi-item measures. Third, educational and occupational groups may differ in both their likelihood and intensity of exposure to communication-tool-mediated work scheduling. Because the focal measure is binary, the available data cannot determine whether such differences contribute to the observed SES-related subgroup patterns. Future studies should examine digital communication practices, temporal autonomy, and boundary control across socioeconomic groups in greater detail.

## Supporting information

S1 Appendix Supplementary analyses and robustness checks. Document containing supplementary materials for the study, including the analytic sample construction, full regression models with control variables, ordered logit and ordered probit estimates, Heckman two-step sample selection results, subgroup analysis by phone on-call status, and supplementary mediation analyses.
(DOCX)
