# Supplementary material for: Work scheduling through communication tools and job satisfaction: The roles of work-family conflict and perceived work pressure
Source: PLoS One. 2026 Jul 6;21(7):e0352350. doi: 10.1371/journal.pone.0352350 (PMC13336460; doi:10.1371/journal.pone.0352350)
Supplement: S1 Appendix — Document containing supplementary materials for the study, including the analytic sample construction, full regression models with control variables, ordered logit and ordered probit estimates, Heckman two-step sample selection results, subgroup analysis by phone on-call status, and supplementary mediation analyses. (DOCX) [file pone.0352350.s001.docx]

**Appendix**

| **Table A1.** Steps of analytic sample construction. | | |
| --- | --- | --- |
| **Steps** | **Sample restriction** | **Sample** |
| 0 | Original sample of CGSS 2021 | N=8,148 |
| 1 | Restricting the sample to respondents who answered the question: “In the past month, has your work been scheduled at any time via WeChat or phone?” | N=1,973 |
| 2 | Restrict the sample to individuals who completed all key questions (excluding those who choose “Not applicable” or “Don’t know”). | N=1,576 |
| 3 | Considering China’s statutory retirement age, restrict the sample to males under 65 years old and females under 60 years old. | N=1,504 (final analytic sample) |

| **Table A2.** Full models including control variables. | | | | |
| --- | --- | --- | --- | --- |
|  | Job satisfaction | | | |
|  | Model 1 | Model 2 | Model 3 | Model 4 |
| Independent variable |  |  |  |  |
| Communication-tool-mediated work scheduling | -0.086** | -0.100** | -0.115** | -0.117** |
|  | (0.043) | (0.048) | (0.057) | (0.058) |
| Control variables |  |  |  |  |
| Gender (Ref. = Male) |  |  |  |  |
| Female |  | 0.076* | 0.100** | 0.100** |
|  |  | (0.043) | (0.046) | (0.048) |
| Age |  | 0.017*** | 0.017*** | 0.017*** |
|  |  | (0.003) | (0.003) | (0.006) |
| Age squared |  | 0.000** | 0.000* | 0.000 |
|  |  | (0.000) | (0.000) | (0.000) |
| *Hukou* type (Ref. = Agricultural) |  |  |  |  |
| Non-Agricultural |  | -0.087 | -0.089 | -0.088 |
|  |  | (0.055) | (0.060) | (0.061) |
| Others |  | -0.094* | -0.078 | -0.077 |
|  |  | (0.053) | (0.059) | (0.061) |
| Education (Ref. = Non-tertiary) |  |  |  |  |
| Tertiary and above |  | 0.137** | 0.130** | 0.132** |
|  |  | (0.055) | (0.059) | (0.060) |
| Marital status (Ref. = Never married) |  |  |  |  |
| Married/cohabiting |  | -0.012 | -0.058 | -0.055 |
|  |  | (0.097) | (0.115) | (0.116) |
| Divorced/separated/widowed |  | -0.207* | -0.264* | -0.255* |
|  |  | (0.125) | (0.146) | (0.150) |
| Union member (Ref. = No) |  |  |  |  |
| Yes |  | -0.006 | -0.023 | -0.030 |
|  |  | (0.056) | (0.062) | (0.063) |
| Parenthood status (Ref. = No) |  |  |  |  |
| Yes |  | 0.043 | 0.057 | 0.059 |
|  |  | (0.092) | (0.111) | (0.112) |
| Party membership (Ref. = No) |  |  |  |  |
| Yes |  | 0.080 | 0.090 | 0.080 |
|  |  | (0.060) | (0.062) | (0.065) |
| Working hours per week |  | -0.009*** | -0.010*** | -0.011*** |
|  |  | (0.001) | (0.002) | (0.002) |
| Working years in present job |  | 0.002 | 0.001 | 0.001 |
|  |  | (0.003) | (0.003) | (0.003) |
| Occupation (Ref. = Professional) |  |  |  |  |
| Non-professional |  | -0.057 | -0.055 | -0.060 |
|  |  | (0.046) | (0.048) | (0.048) |
| Region type (Ref. = Western) |  |  |  |  |
| Northeast |  | 0.257*** | 0.328*** | 0.337*** |
|  |  | (0.097) | (0.104) | (0.107) |
| Central |  | 0.113* | 0.141* | 0.158** |
|  |  | (0.066) | (0.077) | (0.078) |
| East |  | 0.106* | 0.136* | 0.144** |
|  |  | (0.060) | (0.071) | (0.073) |
| Constant | 3.721*** | 3.229*** | 3.326*** | 3.320*** |
|  | (0.037) | (0.201) | (0.251) | (0.373) |
| Observations | 1,967 | 1,576 | 1,576 | 1,504 |
| R-squared | 0.002 | 0.082 | 0.093 | 0.088 |
| *Note*. Standard errors in parentheses. * p<0.1, ** p<0.05, *** p<0.01 | | | | |

| **Table A3.** The association between communication-tool-mediated work scheduling and job satisfaction: Ordered logit and ordered probit estimates | | | | |
| --- | --- | --- | --- | --- |
| Panel A. OLogit | | | | |
|  | Model 1 | Model 2 | Model 3 | Model 4 |
| Communication-tool-mediated work scheduling | -0.162^*^ | -0.207^*^ | -0.244^*^ | -0.241^*^ |
|  | (0.0970) | (0.116) | (0.139) | (0.142) |
| Control variables | No | Yes | Yes | Yes |
| Observations | 1967 | 1576 | 1576 | 1504 |
| Panel B. OProbit | | | | |
|  | Model 1 | Model 2 | Model 3 | Model 4 |
| Communication-tool-mediated work scheduling | -0.111^**^ | -0.140^**^ | -0.163^**^ | -0.163^**^ |
|  | (0.0553) | (0.0651) | (0.0760) | (0.0777) |
| Control variables | No | Yes | Yes | Yes |
| Observations | 1,967 | 1,576 | 1,576 | 1,504 |
| *Note*. Model 2, 3, and 4 control for gender, age, age squared, *hukou* type, educational level, marital status, parenthood status, union status, party membership, region type, occupation, working hours per week, working years in current job. Standard errors in parentheses. * p<0.1, ** p<0.05, *** p<0.01 | | | | |

| **Table A4.** Heckman two-step sample selection results | | | | |
| --- | --- | --- | --- | --- |
|  | Model 1 | Model 2 | Model 3 | Model 4 |
| Communication-tool-mediated work scheduling |  | -0.101** |  | -0.108** |
|  |  | (0.048) |  | (0.050) |
| Evening interview^^[[1]](#footnote-0)^^ | 0.216*** |  | 0.229*** |  |
|  | (0.072) |  | (0.074) |  |
| Control variables | Yes | Yes | Yes | Yes |
| Constant | 0.201 | 3.113*** | 0.031 | 3.070*** |
|  | (0.275) | (0.258) | (0.395) | (0.332) |
| Lambda | 0.201 | | 0.211 | |
|  | (0.279) | | (0.283) | |
| Rho^[[2]](#footnote-1)^ | 0.248 | | 0.258 | |
| Sigma | 0.812 | | 0.816 | |
| Observations | 2,252 | | 2,146 | |
| *Note*: Models 1 and 3 present the first-stage selection equations, while Models 2 and 4 present the second-stage outcome equations. Models 1 and 2 are estimated using the full sample, whereas Models 3 and 4 exclude respondents who have reached statutory retirement age. In the first-stage selection equation, the dependent variable is a dummy variable indicating whether the respondent answered the question on communication-tool-mediated work scheduling. Evening interview is used as the exclusion restriction and is included only in the first-stage selection equation. All models control for gender, age, age squared, *hukou* type, educational level, marital status, parenthood status, union status, party membership, region type, occupation, working hours per week, and working years in the current job. Standard errors are reported in parentheses. * p<0.1, ** p<0.05, *** p<0.01. | | | | |

| **Table A5.** The association between communication-tool-mediated work scheduling and job satisfaction by phone on-call status | | | | | | | |
| --- | --- | --- | --- | --- | --- | --- | --- |
|  | On-call by phone | | |  | Not on-call by phone | | |
|  | Model 1 | Model 2 | Model 3 |  | Model 4 | Model 5 | Model 6 |
| Communication-tool-mediated work scheduling | -0.069 | -0.028 | 0.015 |  | -0.157** | -0.179** | -0.180** |
|  | (0.099) | (0.121) | (0.127) |  | (0.066) | (0.075) | (0.076) |
| Observations | 929 | 929 | 891 |  | 632 | 632 | 600 |
| R-squared | 0.114 | 0.125 | 0.117 |  | 0.083 | 0.088 | 0.085 |
| *Note.* Model 1-6 control for gender, age, age squared, *hukou* type, educational level, marital status, parenthood status, union status, party membership, region type, occupation, working hours per week, working years in current job. Standard errors in parentheses. * p<0.1, ** p<0.05, *** p<0.01 | | | | | | | |

| **Table A6.** The supplementary mediation analysis | | | | | | | | | |
| --- | --- | --- | --- | --- | --- | --- | --- | --- | --- |
| Panel A. Two-/Three-step mediation tests | | | | | | | | | |
|  | WFC | | | |  | Perceived work pressure | | | |
|  | Job satisfaction | WFC | | Job satisfaction |  | Job satisfaction | Perceived work pressure | | Job satisfaction |
| Communication-tool-mediated work scheduling | -0.117** | 0.350*** | | -0.032 |  | -0.117** | 0.267*** | | -0.056 |
|  | (0.058) | (0.049) | | (0.058) |  | (0.058) | (0.061) | | (0.057) |
| WFC | - | - | | -0.257*** |  | - | - | | - |
|  | - | - | | (0.032) |  | - | - | | - |
| Perceived work pressure | - | - | | - |  | - | - | | -0.239*** |
|  | - | - | | - |  | - | - | | (0.028) |
| Observations | 1,504 | 1,500 | | 1,498 |  | 1,504 | 1,502 | | 1,500 |
| R-squared | 0.088 | 0.151 | | 0.140 |  | 0.088 | 0.130 | | 0.152 |
| Panel B. Sobel tests | | | | | | | | | |
| Indirect effect | -0.080*** | | 72.73% | |  | -0.054*** | | 47.79% | |
|  | (0.015) | |  | |  | (0.014) | |  | |
| Direct effect | -0.030 | |  | |  | -0.059 | |  | |
|  | (0.050) | |  | |  | (0.049) | |  | |
| Total effect | -0.110** | |  | |  | -0.113** | |  | |
|  | ( 0.050) | |  | |  | (0.050) | |  | |
| Observations | 1,498 | |  | |  | 1,500 | |  | |
| Panel C. Bootstrap tests (with 1,000 replications) | | | | | | | | | |
| Indirect effect | -0.080*** | | | |  | -0.054*** | | | |
|  | (0.014) | | | |  | (0.014) | | | |
| Direct effect | -0.030 | | | |  | -0.059 | | | |
|  | (0.050) | | | |  | (0.049) | | | |
| Observations | 1,498 | | | |  | 1,500 | | | |
| *Note.* The purpose of this analysis is to help illuminate the empirical pattern of associations among work scheduling through communication tools, WFC, perceived work pressure, and job satisfaction, rather than to establish a strict mediating mechanism. The supplementary analyses examine WFC and perceived work pressure separately and are intended to assess the robustness of the presence and direction of each statistical pathway. They do not estimate the same joint multiple-mediator quantity as the KHB decomposition, and their indirect-effect magnitudes should therefore not be compared directly with the mediator-specific contributions reported in Table 3. All panels control for gender, age, age squared, *hukou* type, educational level, marital status, parenthood status, union status, party membership, region type, occupation, working hours per week, working years in current job. Standard errors in parentheses. * p<0.1, ** p<0.05, *** p<0.01 | | | | | | | | | |

1. Evening interview is defined as an interview conducted at 18:00 or later. Interview timing may affect item response by influencing respondents’ fatigue, patience, and questionnaire completion. We use this survey-process variable as an exclusion restriction because it is expected to be more directly related to respondents’ likelihood of answering the focal survey item than to their underlying job satisfaction, conditional on the observed individual and work-related covariates. Additional analyses support the plausibility of this assumption. Evening interview timing significantly predicts item response in both the full sample and the sample excluding respondents above statutory retirement age, but it is not significantly associated with job satisfaction in either sample. Moreover, when evening interview timing is included in both the selection and outcome equations, the estimated coefficient of communication-tool-mediated work scheduling remains unchanged. Nevertheless, because the exclusion restriction cannot be definitively verified, the Heckman analysis is interpreted only as a supplementary robustness check. [↑](#footnote-ref-0)
2. The estimated rho values are positive in both specifications, indicating that the correlation between the unobserved factors affecting response to the communication-tool-mediated work scheduling item and those affecting job satisfaction is positive in direction. However, the inverse Mills ratio is not statistically significant, suggesting that there is no strong evidence of substantial sample selection bias. Therefore, the Heckman results are better interpreted as a robustness check, indicating that the main conclusions are not materially affected by sample selection concerns. [↑](#footnote-ref-1)
